# Understanding Factors Influencing Dog Owners' Intention to Vaccinate Against Rabies Evaluated Using Health Belief Model Constructs

**DOI:** 10.3389/fvets.2018.00159

**Published:** 2018-07-11

**Authors:** Tariku Jibat Beyene, Beakal Mindaye, Samson Leta, Natalia Cernicchiaro, Crawford W. Revie

**Affiliations:** ^1^College of Veterinary Medicine and Agriculture, Addis Ababa University, Bishoftu, Ethiopia; ^2^Department of Diagnostic Medicine and Pathobiology, College of Veterinary Medicine, Center for Outcomes Research and Epidemiology, Kansas State University, Manhattan, KS, United States; ^3^Centre for Veterinary Epidemiological Research, Atlantic Veterinary College, University of Prince Edward Island, Charlottetown, PE, Canada

**Keywords:** Ethiopia, health belief model, ordinal regression model, rabies, vaccination

## Abstract

Ethiopia has one of the highest incidence levels of human rabies in Africa, with 3–7 deaths per 100,000 people annually. The country has no official rabies control programme, despite the availability of an effective canine vaccine to control rabies. To support effective rabies control, an understanding of the factors affecting dog owners' voluntary intentions to vaccinate their dogs is important. As such, this study examined factors influencing dog owners' intentions to vaccinate their dogs using the constructs of health belief theory. In this cross-sectional study, a questionnaire, designed based on the Health Belief Model constructs was completed by 249 dog owners in 9 randomly selected wards of Bishoftu town in central Ethiopia between October and December 2016. An ordinal regression model was then fitted to explore factors which best predict the likelihood of a dog owner's intention. A classification and regression tree (CART) model was then used for recursive partitioning of the Likert scale in the significant variables to distinctively classify ordinal categories of vaccination intention. Participants' preventive intention was associated with the six constructs of the Health Belief Model: perceived susceptibility, readiness to action, self-efficacy, perceived threat, benefits, and barriers. Dog owner's knowledge about rabies was found to be positively associated with intention to vaccinate, whereas distance from vaccination centers and difficulty of dog transportation were found to be negatively associated to intention to vaccinate. Distance from vaccination center was found to be the best predictor for the intention to vaccinate. The results of this study have policy implications for controlling rabies including increasing dog owners' knowledge about rabies, locating vaccination centers at shorter distances from dog populations and providing suitable means to transport dogs to vaccination centers.

## Introduction

Rabies is a viral disease caused by a negative-stranded RNA virus of the genus *Lyssavirus* in the family *Rhabdoviridae*, order *Mononegavirales* (1). It is a fatal disease largely transmitted to humans by bites from infected animals—predominantly from domestic dogs. Globally canine-mediated rabies causes about 60,000 human deaths/ per year, of which 24,000 are contributed by African cases. The largest proportion of the estimated economic loss of 8.6 billion USD per year is due to premature death, followed by direct costs incurred in post-exposure prophylaxis and lost income whilst seeking post-exposure prophylaxis, with only limited costs to the veterinary sector due to dog vaccination, and additional costs due to livestock losses (2).

The most effective strategy available to prevent rabies in humans and livestock is preventive vaccination of dogs (3, 4). The World Health Organization also recommends dog vaccination as well as preventive immunization of people with high-risk exposure to rabies. The effectiveness of rabies control through dog vaccination relies on vaccinating a sufficient proportion of the dog population (5).

The success of rabies elimination in dogs through vaccination depends on the commitment and collaboration of the stakeholders involved (6). In Ethiopia, there is no official rabies control program enforced yet and dog vaccination coverage is about 20% in urban areas and non-existent in a rural area (7, 8). In urban areas, where private and public veterinary clinics are available, few dog owners voluntarily vaccinate their dog at their own cost; while in rural areas where private and public veterinary clinics are generally not available, dog owners have no opportunity to get their dogs vaccinated. Similar studies done in developing countries where canine rabies is endemic have shown that lack of knowledge on the burden of the disease and its prevention, perceived high vaccination cost, and easiness to catch unrestrained dogs for vaccination are the most common reasons for dog owners not to vaccinate their dogs (9–11).

Individuals' perception of disease risk is viewed as a fundamental element of most theoretical models of public health and risk perception-behavior (12). Similarly, several theories have been applied to study zoonotic disease risk perception and public disease protective behaviors (13, 14). The Health Belief Model (HBM) is one of the most applied conceptual frameworks in health behavior research and maintenance of health-related behavior. It is a guiding framework for health behavior interventions including personal beliefs or perceptions about a disease and the strategies available to decrease its occurrence (15–18).

Regresion analysis have been widely used to understand which independent variables are related to the dependent variable, and to explore the forms of relationships. In addition, special cases like classification and regresion tree (CART) have been in use for the pictorial presentations that are relatively simple for non-statisticians to interpret (19).

Studies have been conducted in Ethiopia (20–22) to explore knowledge, attitude, practice and socio-demographic factors influencing rabies control. However, the link between these factors and dog owners' intention to vaccinate against rabies remain unclear. Moreover, we believe that more insights are needed regarding protective behaviors explained by psychological factors. This study aims to identify factors influencing dog rabies vaccination behavior among dog owners in the urban district of Bishoftu in Ethiopia using constructs of the Health Belief theory.

## Methods

### Study participants and study design

This was a cross-sectional study conducted in the town of Bishoftu located in central Ethiopia between October and December 2016. The town of Bishoftu has 9 sub-town divisions. Each sub-town was divided into approximately equal sized sub-town wards enclosed by major roads. Data on major roads were obtained from a Google satellite map and their current status was confirmed by visits to the relevant wards. One ward was randomly selected from each sub-town. From each selected ward, only the first 30 households that owned a dog were included in the study mainly for logistics reasons. Dog owners were interviewed using a structured questionnaire translated in local language called Amharic or Oromifa (as needed) addressing levels of knowledge on rabies and preventions options. As we were not certain about the level of literacy of the study participants, a structured questionnaire was administered in the form of an interview after oral consent had been obtained.

### Questionnaire

We developed items for the questionnaire following a modified Health Belief Model (HBM) constructs approach, used to explore predictors of the owner's intention to have their dog vaccinated following Rosenstock, 1974 (12) and in a similar manner to previous research elsewhere (17, 23, 24). The HBM includes six key constructs which influence health protection behaviors: knowledge, perceived threat, perceived benefits, perceived barriers, readiness to action and self-efficacy. Perceived threat included both susceptibility and severity about the risk of being exposed to rabies and concerns around the seriousness of the illness, respectively. Perceived benefits relate to the outcomes that reduce susceptibility and/or severity. Perceived barriers identify concerns or negative beliefs about the intended protective behavior. Readiness to action are strategies or information sources that promote the adoption of the protective behavior. Self-efficacy measures the dog owners' confidence in their ability to adopt the behavior. General socio-economic (income/household spending, educational level), demographic (age, gender), and rabies exposure-related factors (bite history with in a family, health professional friend of the family, if the family vaccinated their dog the current year) were also captured in the questionnaire (details of the list with categories with in each factors/variables is shown in Table 1). Since estimating income data was difficult, we asked about the daily overall spending of each dog owner as a proxy estimate of their income. The questionnaire was pre-tested by administering it to 30 household owners from one ward. Based on these pre-tests, the questionnaire was adjusted mainly for language and clarity. Since the response on the intention to vaccinate dogs could be undecided, to capture the various degrees of intention, the Likert scale of 1–5 was used (17). The questionnaire used is provided as Supplementary Material.

**Table 1:** Comparison of sociodemographic, and rabies related factors across intention to vaccinate (*P*-values indicate significant differences in proportions of categories of variables).

**Variables**	**Category**		**Intention to vaccinate dog/s (percentage)**			
		***n***	**Unlikely (*n* = 78)**	**Somewhat likely (*n* = 20)**	**Likely (*n* = 151)**	***P*-value**
Age	<30	10	0.20	0.20	0.60	<0.01
	31–40	91	0.53	0.08	0.40	
	41–50	87	0.25	0.08	0.67	
	51–60	46	0.13	0.07	0.80	
	>61	15	0.00	0.07	0.93	
Gender of the respondents	Male	98	0.34	0.08	0.58	0.79
	Female	151	0.30	0.08	0.62	
Educational level of the respondents	No formal education	22	0.05	0.05	0.91	0.03
	Elementary	38	0.18	0.11	0.71	
	High school	121	0.36	0.09	0.55	
	College	68	0.40	0.06	0.54	
Income/Household spending (USD/day)	<1 USD	49	0.35	0.12	0.53	0.47
	1–5 USD	103	0.25	0.08	0.67	
	6–10 USD	70	0.39	0.06	0.56	
	>11 USD	27	0.30	0.07	0.63	
Have health professional or veterinarian friend or relative	Yes	41	0.02	0.05	0.93	<0.01
	No	208	0.37	0.09	0.54	
Family member of self ever been bitten by suspected rabid dog	Yes	27	0.00	0.15	0.85	<0.01
	No	222	0.35	0.07	0.58	
Vaccinated your dog this year	Yes	95	0.00	0.11	0.89	<0.01
	No	154	0.51	0.06	0.43	

#### Dog rabies vaccination intention

Four variations of questions relating to the key outcome were used to assess likelihood of future vaccination: (1) did the owner intend to have their dog vaccinated in the next 12 months?; (2) did the owner have a plan as to how and where they would have their dog vaccinated?; (3) what was the strength of the owner's intention to have their dog vaccinated the following year?; and (4) how likely was it that they would get their dog be vaccinated in the coming year? For each question, responses were recorded on a 5-point Likert scale ranging from “*Definitely not/highly unlikely*” to “*Definitely/highly likely*.”

#### Knowledge

To assess the knowledge construct, three components were considered, namely: knowledge on the cause of rabies, general consequences of the disease and identification of dogs that could transmit rabies. Three questions were asked to assess knowledge of the causes of rabies (e.g., “How can a person be exposed to rabies? Options for answering this question consisted of 1 = through a bite, 2 = through a scratch, 3 = don't know). Likewise, four questions were asked to assess knowledge of potential consequences, and two questions were asked to assess knowledge on how to recognize a rabid animal that could transmit the disease based on yes or no option to answer. Then, correct answer to each question was given 1, otherwise 0. For each of the three components of the construct knowledge namely: knowledge on the cause of rabies, general consequences of the disease and identification of dogs that could transmit rabies, the response to each question was summed up to get score for each of the components of knowledge construct.

#### Perceived threat

Two categories of threat were assessed, namely perceived susceptibility and severity. Two questions were asked for each category, with a question on susceptibility (e.g., without rabies vaccine, what do you think the likelihood is that yours'/neighbor's dog can get rabid at some point in the future?) followed by a closely related question on severity (e.g., Would it be serious for you, your family and/or neighbors if your dog became infected with rabies”?). Responses were recorded on a 5-point scale ranging from “very unlikely” to “very likely” and “strongly disagree” to “strongly agree” for perceived susceptibility and severity components, respectively.

#### Perceived benefits

Three questions were used as indicators of perceived benefits of using rabies dog vaccination. For instance, “If I get my dog vaccinated next year, I will decrease the chance of rabies to myself/family and neighbors” was scored on a scale ranging from strongly disagree to strongly agree.

#### Perceived barriers

Five key questions were used as indicators to the barriers to rabies dog vaccination which included distance to vaccination centers, the cost of vaccination, ease of dog transportation, ease of handling dogs, and trust in the vaccine. These barriers were assessed with one question each on a 5 point scale from “strongly disagree” to “strongly agree.” For instance, for the barrier related to distance to vaccination centers we asked the following: “If I want to vaccinate my dog, vaccination centers are not far from my house.”

#### Readiness to action

Four key questions were asked to assess the level of readiness to act on dog rabies vaccination information including their enthusiasm to seek information on the disease or vaccination status of their dogs. For instance, “I look for information about rabies in general and I am likely to stop, read and think about it when I encounter information about rabies” on a five-point scale from “strongly disagree” to “strongly agree.”

#### Self-efficacy

Self-efficacy of the dog owners' ability to get their dogs to vaccinated were measured by three questions related to the location where and when to vaccinate their dogs (e.g., If I want to vaccinate my dog, I know where and when to vaccinate my dog). Responses were reported on a 5-point Likert scale ranging from “strongly disagree” to “strongly agree.”

### Data analysis

Data were collected in a structured table and analyzed using the R statistical package. The purpose of the study was to examine factors influencing dog rabies vaccination intention-behavior using constructs of the Health Belief Model. For each construct in the model, multiple questions (hereafter called items) were posed to check for internal consistency using Cronbach's alpha (25) available in the R package “psy”. Cronbach's alpha was used as a measure of internal consistency, that is, how closely related a set of items are as a group named hereafter construct (26). Using the function Cronbach, whenever the internal consistency among items within a construct was greater or equal to 0.7, the average Likert score for that construct was calculated. If the internal consistency among questions within a construct was lower than 0.7 then each item was treated separately. However, for the case of knowledge where the internal consistency among the scores of three components of the knowledge construct was less than 0.7, we summed up the scores of the three components to get overall score for knowledge construct.

Within the health belief model, the intention to adopt protective behavior is considered to be the primary dependent variable, while other constructs can have direct or indirect explanatory value (27). Looking at the distribution of the averaged 5-point Likert scale for preventive intention, the outcome variable was categorized into three groups: average value of ≤2.75 as “Unlikely” to vaccinate, 2.75 to ≤3.5 as “Somewhat likely” to vaccinate and >3.5 as “Very likely” to vaccinate.

A univariable analysis was conducted to evaluate associations between groups of socio-economic, demographic and rabies exposure-related factors with the intention to vaccinate using Pearson's Chi-squared test. Significance was tested at *P* < 0.05.

Correlation matrices for each of the HMB constructs were calculated using the Cronbach function using the R statistical software. The internal consistency for four constructs namely, perceived threats, perceived benefits, readiness to action and self-efficacy indicated Cronbach alpha of 0.7 for the underlying items (questions) and arithmetic average of the Likert scale of the item were used to represent the construct. However, for underlying items (questions) describing perceived barriers and the scores for three components of knowledge, the Cronbach alpha was below 0.7. As a result, items relating to perceived barriers were treated separately but single score for the knowledge construct was generated by summing up the scores of the three components of knowledge. Then the variables perceived threats, perceived benefits, readiness to action, self-efficacy, items under perceived barriers (namely distance from vaccination center, cost of vaccine, ease of transportation dogs, east of capturing dogs, and trust on vaccine), and knowledge were selected to be candidates for inclusion in the final multivariable ordinal logistic regression model.

#### Model building

The strategy followed to build the final multivariable ordinal logistic regression model consisted of (1) screening variables and estimating correlation among HBM construct candidate variables (i.e., knowledge, perceived susceptibility, readiness to action, self-efficacy, perceived threat, benefits, and barriers: variables with correlation coefficients greater than |0.7| were removed (theoretical background was considered for the selection), (2) creating and evaluating all possible 2-way interaction terms, (3) selecting variables and interaction terms which are significant and including them in the maximal multivariable ordinal model, (4) checking for presence of confounding by comparing if the direction or significance of relationship between the predictor variables and the outcome variable changed, and (5) the variance inflation factor (VIF) was used to examining the multicollinearity among the variables: VIF values greater than or equal to 10 were assumed to indicate collinearity. The fit of the final model was assessed using the McFadden pseudo-*R*^2^ to assess predictive power, with McFadden pseudo-*R*^2^ preferred value range of 0.2–0.4 and likelihood-ratio tests to compare the final saturated model with the null model.

Further, a classification and regression tree (CART) model was used for recursive partitioning analysis using the “rpart” package in R software version 3.0.2. The CART method was applied to the different categories of the significant variables identified during the ordinal regression modeling to construct a decision tree composed of progressive binary splits of the predictive variables identified by the ordinal regression model. Each parent node in the decision tree produces two child nodes, which in turn can become parent nodes producing additional children. The process continues, and includes both tree building and pruning until statistical analysis indicates that the tree adequately fits the information contained in the dataset (28).

## Results

### Study participants and study design

During the field survey, the study team was able to interview 30 dog owning adult household heads in four wards; namely wards 2, 4, 5, and 6. In two of these, wards 1 and 3, one questionnaire was incomplete from each ward. In the remaining three wards, some households were not interviewed as there was no adult household head to respond. Hence, a total of 249 households were surveyed.

### Characteristics of socio-economic and demographic variables across dog owners across intention

A comparison of dog owners' socioeconomic, geographic and rabies exposure-related factors across the categories of the intention to vaccinate is given in Table 1. There were no statistically significant differences between proportions in the three “intention to vaccinate” categories for the variables gender of the respondent (*P* = 0.79), and daily household spend/income (*P* = 0.47). In this study, older dog owners are more likely to vaccinate their dogs compared to younger owners (*P* < 0.01). Surprisingly, owners with higher educational level were shown to have less likely intention compared to those with lower educational level (*P* = 0.03). Dog owners who have health professionals or veterinarians as relative/friend, family member/relatives that have ever been bitten by a suspected dog, and those who have vaccinated their dogs this year, were found to be more likely to intend to vaccinate their dogs (*P* < 0.01) (Table 1).

### Internal consistency and correlation coefficients of the constructs

The level of internal consistency for items included under the constructs of vaccination intention (outcome), perceived threats, perceived benefits, readiness to action, and self-efficacy was all greater than 0.7. Accordingly, we averaged the Likert scaled responses for these constructs. For the case of the constructs: perceived barriers and knowledge the internal consistency among items within a construct was lower than 0.7 and each item was treated as a separate variable within these constructs.

For the knowledge, there was poor internal consistency (Cronbach's alpha = 0.11) among the three variables i.e., cause, consequence, and identification. Accordingly, based on literature, appropriate responses to cause, consequence and knowledge on rabies received a value of 1, while inappropriate or “don't know” responses received a value of 0. Therefore, depending on the total number of items each category ranged from 0 to the number of items under each component.

The internal consistency for each construct was estimated to be as follows: Knowledge (0.11), preventive intention to vaccinate dogs (0.98), perceived benefit (0.99), barrier (-0.05), threat (0.93), readiness to action (0.73), and self-efficacy (0.96).

The average Likert points, SDs, minimum/maximum scores, and correlation coefficients between all constructs and measured variables are given in Table 2. The average score on the protective intention was 2.29 on a 5 points in the Likert scale () (Table 2). Bivariable correlation analyses provided in Table 2 indicate that intention to vaccinate their dogs against rabies through vaccination is positively associated with overall knowledge, perceived benefits, trust in the vaccine, perceived threat, readiness to act, and self-efficacy. However, it is negatively associated with perceived barriers such as distance from the vaccination center, and the need to transport dogs. There was a very weak association to perceived barriers such as the cost of vaccination and the ease of capturing dogs.

**Table 2:** Mean, SD, and correlations coefficients between all constructs and measured variables.

	**Mean**	**SD**	**Min-Max**	**Protective intention**	Knowledge^*^	Benefits^*^	Distance^*^	Cost^*^	Ease of transporting^*^	Ease of capturing^*^	Trust^*^	Threat^*^	Cue to action^*^	Self-efficacy^*^
Protective intention	2.29	0.91	1–3	1.00										
Knowledge	6.4	0.63	4–8	0.43	1.00									
Perceived benefits	3.37	0.93	2–4	0.95	0.44	1.00								
Perceived barrier														
Distance	2	0.74	1–3	−0.71	−0.28	−0.76	1.00							
Cost of vaccine	1	0.06	1–2	0.05	−0.04	0.04	0.00	1.00						
Ease of transporting dogs	2.1	0.53	1–3	−0.36	−0.11	−0.36	0.42	0.11	1.00					
Ease of capturing dogs	1.59	0.53	1–3	−0.18	−0.12	−0.18	0.26	0.05	0.19	1.00				
Trust in the vaccine	3.73	0.52	2–5	0.74	0.32	0.75	−0.57	0.03	−0.27	−0.20	1.00			
Perceived threat	4.09	0.68	2.7–4.7	0.90	0.41	0.93	−0.71	0.04	−0.37	−0.16	0.76	1.00		
Readiness to action	2.3	0.43	1.3–3	0.84	0.36	0.87	−0.73	−0.01	−0.43	−0.22	0.69	0.83	1.00	
Self-efficacy	3.61	0.59	2–4	0.92	0.48	0.96	−0.74	0.04	−0.40	−0.17	0.76	0.93	0.85	1.00

* *Horizontal heading was shortened version of the vertical headings*.

### Dog owner's intentions to vaccinate their dogs

The ordinal logistic regression was run with candidate variables being: perceived threats, perceived benefits, readiness to action and self-efficacy constructs, the overall (sum) of knowledge, and the specific items relating to perceived barriers (i distance from vaccination center, cost of vaccine, ease of transportation dogs, east of capturing dogs, and trust on vaccine). Only three variables remained significant in the final model (1) knowledge, (2) distance from vaccination center, and (3) ease of transporting (Table 3).

**Table 3:** Model predictors for dog owners' intention to vaccinate their dogs, showing coefficients, significance values, and odds ratios with 95% CI.

	**Coefficient**.	**S.E**.	***t*-value**	***P*-value**	**OR [OR 95% CI]**
Knowledge	1.32	0.31	4.25	<0.01	3.74 [2.08–7.05]
Perceived barrier:					
Distance from	−2.39	0.33	−7.31	<0.01	0.09 [0.05–0.17]
vaccination center					
Ease of	−0.71	0.36	−1.97	0.05	0.49 [0.24–0.98]
transporting dogs				
Ease of	0.11	0.33	0.33	0.74	1.12 [0.59–2.16]
capturing dog					

*Model fitted was knowledge, perceived barriers: Distance from vaccination center, Ease of transporting dogs, and ease of capturing dog with intention to vaccinate*.

The intention to vaccinate dogs was positively associated with knowledge while negatively associated with distance from vaccination center and ease of transporting dogs. For knowledge which is treated as a continuous variable, when a dog owner's knowledge moves up by 1 unit, the odds of moving from “unlikely” to “somewhat likely” or “very likely” intention to vaccinate dogs increase 3.74 times. For the categorical variables (measured in a 5 point Likert scale), for each one unit increase in response from “strongly disagree” to “strongly agree” for the question, “If I want to vaccinate my dog, vaccination centers are not far from my house and reduces the chance for me to get my dog vaccinated,” the odds of “very likely” intention vs. “somewhat likely” or “unlikely” intention increase by 11 times (1/0.09), given that all of the other variables in the model are held constant. Likewise, for the question “If I want to vaccinate my dog, transportation is easy to get my dog to vaccination center,” as the response goes from “strongly disagree” to agree and then to “strongly agree,” the odds of “very likely” or “somewhat likely” intention vs. “unlikely” intention increase 2 times (1/0.49). The fitted ordinal model had acceptable predictive value (McFadden Pseudo *R*^2^ = 0.37) and the likelihood-ratio comparing the full model to the null model was found to be significant (*P* < 0.01). The model summary of this analysis is shown in Table 3.

### Stratification of predictor variables

**Figure 1:**
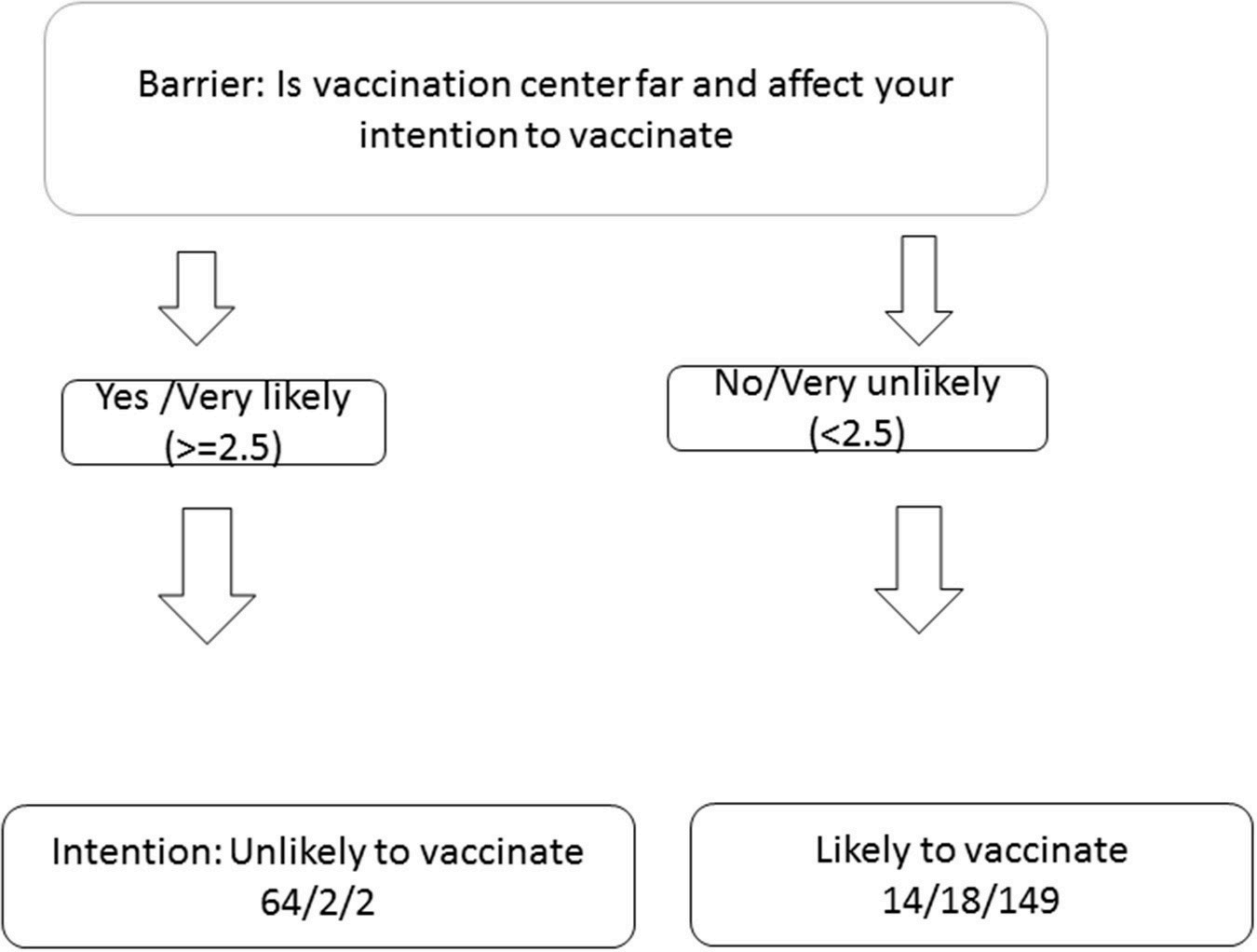
Classification tree for preventive behavior. Numbers in bracket indicate those stating that they will not vaccinate, maybe vaccinate and will vaccinate their dogs respectively. For instance, about unlikely to vaccinate: 82% (64/78), somehow likely to vaccinate 10% (2/20) and likely to vaccinate 1% (2/151) of dog owners who were categorized as unlikely, somewhat likely and likely to vaccinate respectively indicated agreement (≥2.5) to the question: “If I want to vaccinate my dog, vaccination centers being located far from my house reduce the chance for me to get my dog vaccinated.” Similarly, 18% (14/78), 90% (18/20), and 99% (149/151) of dog owners who were categorized as unlikely, somewhat likely and likely to vaccinate respectively responded strongly disagree or disagree to the question.

The decision tree generated by CART was tested for its ability to stratify the three levels of protective intention. Variables which were significant in the ordinal regression model were included in the CART model. Thus, the three variables: knowledge, the perceived barrier variables of distance to vaccination center and ease of dog transportation were included. Using protective intention as the outcome, the CART was able to stratify dog owners' intention into two distinct groups using only the distance as a barrier variable (Figure 1). Response to the variable distance was found to be the best and only significant predictor of intention to vaccinate. Accordingly, owners who replied to the question “If I want to vaccinate my dog, vaccination centers being located far from my house reduce the chance for me to get my dog vaccinated” using a Likert scale value of greater or equal to 2.5 were correctly categorized for 82% (64/78), 10% (2/20) and 1% (2/151) of cases in the unlikely, somewhat likely and likely categories, respectively. Similarly, for a response of 1 or 2, the categories were 18% (14/78), 90% (18/20), and 99% (149/151) in the same order.

## Discussion

This study aimed at understanding factors influencing rabies preventive intention behaviors as they relate to constructs of the Health Belief Theory within a community of dog owners in the town of Bishoftu, in central Ethiopia. Such method has been used in social epidemiology and health psychology to understanding factors that influence disease preventive measures (15–18, 23, 24).

We found that older dog owners were more likely to vaccinate their dogs compared to younger owners. Despite the fact that there are no similar studies to compare with, previous studies on knowledge on rabies reported better knowledge in older dog owners (24). Conversely, we found lower intention to vaccinate among dog owners with higher educational status. This could be related to the relatively lower sample size in the category of respondent with lower educational level. Kabeta et al. (24) did not find significant difference on knowledge on rabies among respondents of various educational level. We found that owners' intention to vaccinate their dogs were positively associated with the dog owners' knowledge of rabies, perceived benefits, threat, readiness to action, self-efficacy and trust in the vaccination. However, it was found to be inversely related to perceived barriers such as the distance of the owners' residence from vaccination center and ease of dog transportation. Our study is consistent with other similar studies of the Health Belief Theory (17, 23, 24). However, using an ordinal logistic regression model, only the dog owner's knowledge on rabies, distance from the vaccination center and ease of dog transportation were found to be significant predictors for the level of intention to vaccinate their dogs so that rabies can be prevented.

Using the CART method, which aims at stratifying variables based on their importance, the variable distance from vaccination center partitioned the prevention intention variable into two major levels from unlikely to very likely. In this study, we identified the important factors predicting intention to vaccinate dogs using regression model followed by a CART method which stratified the identified variables. Identifying significant factors would drive decisions regarding allocation of preventive measures. Furthermore, the pictorial presentation and flow diagram produced using a CART would be easy to interpret and explain to policymakers.

Many studies have assessed knowledge, attitude and practices of rabies prevention through vaccination using socio-economic variables (21, 29). As human behavior plays a central role in the maintenance of health, and the prevention of disease, in addition to the socio-demographic factors, further insight can be gained on factors influencing dog owner's intention to vaccinate their dogs if psychological factors are explored. Intention could be one of the best predictors of action. One of the earliest theoretical models developed for understanding health behaviors was the health belief model (30). In this study, we found that 60% of the dog owners had a strong intention to vaccinate their dogs, whereas only 38% of these owners had vaccinated their dog this year. This difference between intention and action could be further explored by examining some other factors like psychological factors, amount of time allocated might be required to fully interpret intention. Furthermore, we would expect fewer barriers to vaccination in those who had vaccinated their dogs in the past year compared to those who are only now intending to vaccinate their dogs. However, the comparison is left for further exploration as this was not main objective of the present study.

Irrespective of the difference in scales for measures around the constructed knowledge, the mean score was a little above the midpoint of the range of possible scores. This indicates that dog owners in Bishoftu are currently fairly knowledgeable about rabies. The variable knowledge being one of the predictors of intention indicates that improving knowledge could positively contribute to vaccination intention. The role and importance of knowledge in the formation of positive prevention intention for both infectious and non-infectious diseases have been reported in several studies (31, 32). The level of public knowledge in relation to protective intention has also practical implications for health education, research and the campaign toward dog-mediated rabies elimination led by WHO and FAO (33). The mean score of the constructs of perceived benefit, threat, self-efficacy, and readiness to action was well above the midpoint of the possible range. This is in line with literature reporting that rabies is one of the most feared and important threats to public health (34). In this study, neither cost of dog vaccine nor the daily income of the dog owners were found to have a significant association with vaccination intention. This finding is inconsistent with other reports which have found that the cost of dog vaccination to be one of the major barriers to rabies control (10, 29, 35). Logistics such as distance from vaccination center and difficulty of transporting dogs to vaccination centers are also important negative predictors of intention to vaccinate. Many of vaccination centers in Ethiopia are clustered in urban areas and those in towns are based in public veterinary clinics which are few. Studies also reported that as distance to the central vaccination center increases so vaccination coverage decreases (36). Furthermore, in Africa and elsewhere, it has also been shown that difficulty of transporting dogs to the vaccination sites is a primary reason for non-vaccination during outbreaks (37–39). On the other hand, the CART method indicated that making vaccination available to the “door-to-door” level might not ensure complete coverage, as close to 10% of the dog owners who were categorized in the very unlikely to vaccinate their dogs category, also responded that distance did not matter. Conversely, complete coverage is not a requirement and 70% coverage would be adequate to control rabies (5).

In this study, it was shown that the level of association of the constructs met the expectations of the health belief theory as the protective intention was positively associated to knowledge, readiness to action, self-efficacy, perceived benefit and negatively associated to barriers. Our findings are consistent with findings that indicate perceived barriers are the most powerful predictors of protective intentions (23).

Despite the fact that we adopted a different method than the traditional KAP approach to explore factors influencing dog owners' intention to vaccinate, this study provided only limited novel insights on perceived self-efficacy and threat. Another limitation of the study was its generalizability. The limited number of households sampled, potential varying distribution of dog population within the studied wards, and the nature of the district studied does not give us the liberty to generalize the situation to the whole country of Ethiopia. The district studied, Bishoftu, is one of the towns in the country where relatively good private and public veterinary services, especially for dog rabies vaccination, are available. In contrast, in some urban districts and almost all rural districts, public and private dog vaccination services are generally not available. On the other hand, a study such as this could not be feasibly carried out in rural districts where current vaccination coverage is non-existent and people would have limited awareness as to its purpose. Thus, the findings of this study should be interpreted with caution. Furthermore, the results of this study could have been different had the order of questions posed to dog owners been reversed; i.e., asking about their intention to vaccinate after having covered questions related to severity. Owners may have altered responses regarding their likelihood to vaccinate, had the severity and susceptibility questions been asked beforehand; essentially reminding them to think about how serious rabies could be to them and their family. Similarly, we would expect different responses to questions if they had been structured in an open-ended manner rather than being based on multiple choice or a Likert scale.

In conclusion, we evaluated factors affecting the likelihood of dog owners' intention to vaccinate their dogs against rabies. Our findings indicate that dog owners' intention to vaccinate their dogs is strongly influenced by knowledge, ease of transporting dogs and distance to the vaccination center. The results of this study have policy implications when attempting to control rabies through mass vaccination; in that increasing dog owners' knowledge about rabies, locating vaccination centers close to the target population and facilitating the transportation of dogs to vaccination points, are all likely to improve the success of such voluntary campaigns. Moreover, the results from the classification and regression tree analysis can assist decision makers to understand which variables are the most important ones and what proportion of the people could potentially intend to vaccinate if the identified factors were addressed.

## Ethics statement

This work required neither sampling nor experimentation on animals or humans. However, informed consent was taken from dog owners during data collection. In addition, same administrative wards of Bishoftu town was covered as the previous study approved by the Ethiopian public health institute. The ethical clearance approval letter from the Ethiopian public health institute is provided as supplementary.

## Author contributions

TB conceived the idea of the study. TB, BM, and CR designed the fieldwork, data collection, organized the data, analyzed the data, interpreted results, wrote the manuscript, and approved the version to be published. SL and NC helped in the data analysis, in drafting the manuscript and approved the final version of the manuscript.

### Conflict of interest statement

The authors declare that the research was conducted in the absence of any commercial or financial relationships that could be construed as a potential conflict of interest.
